# Influence of cardiac function on intermittent hypoxia in rats fed with high-fat diet

**DOI:** 10.1016/j.bbrep.2022.101393

**Published:** 2022-11-23

**Authors:** Hideyuki Maeda, Jun Hosomichi, Akihiro Hasumi, Ken-ichi Yoshida

**Affiliations:** a Department of Forensic Medicine, Tokyo Medical University, 6-1-1 Shinjuku, Shinjuku-ku, Tokyo, 160-8402, Japan; b Department of Orthodontic Science, Graduate School of Medical and Dental Sciences, Tokyo Medical and Dental University (TMDU), 1-5-45 Yushima, Bunkyo-ku, Tokyo, 113-8549, Japan

**Keywords:** Sleep apnea syndrome, High-fat diet, Heme oxygenase-1, Echocardiogram, Ejection fraction

## Abstract

A high-fat diet (HFD) accumulates fat in the cardiovascular system, alters the metabolism, and affects cardiac function. Dyslipidemia is associated with the development of sleep apnea syndrome (SAS), which is associated with intermittent hypoxia (IH); however, it is unclear whether SAS affects cardiac function in patients with dyslipidemia. The purpose of this study was to evaluate how IH affects cardiac function in rats fed with a HFD. Male 5-week-old Sprague-Dawley rats of two groups (normal diet (SD) and HFD) were divided into IH-exposed and unexposed groups. Zinc protoporphyrin-9 (ZnPPIX) was administered as a heme oxygenase-1 (HO-1) inhibitor to the SD and IH + HFD groups, and cardiac function and blood viscosity were examined. In the IH + HFD group, echocardiography showed an increased fractional shortening (FS), which peaked on day 4. Western blot analysis revealed an increase in HO-1 after 2 weeks. This peak continued even after the HO-1 inhibitor and ZnPPIX were administered. One cause of increased FS is the stagnation of blood flow due to an increased blood viscosity. To be able to send highly viscous blood to every corner of the body, the heart must contract strongly. Therefore, HO-1 is released by the body as a biological defense reaction. HO-1 has a vasodilatory effect and suppresses hyper constriction. Thus, IH exposure to HFD causes and drives transient hyper constriction, releasing HO-1 as a biological response. This led to dilated blood vessels, after which the FS returned to normal.

## Highlights

• Intermittent hypoxia with high-fat diet causes/drives transient hyper constriction.
• This releases heme oxygenase-1 as a biological response.
• This causes dilated blood vessels, and fractional shortening returned to normal.

## Non-standard abbreviations and acronyms:

CaMKII: Ca2+/Calmodulin-Dependent Protein Kinase II
CRP: C-reactive protein
FS: fractional shortening
HDL-C, high density lipoprotein-cholesterol
HF: heart failure
HFD: high-fat diet;
HO-1: heme oxygenase-1
HR: heart rate
IH: intermittent hypoxia
IL-6: interleukin-6
LDL-C, low density lipoprotein-C; TG, triglycerides
LV: left ventricular
LVAWd LV: anterior wall thickness in diastole
LVAWs LV: anterior wall thickness in systole
LVDd LV: internal dimensions at end diastole
LVDs LV: internal dimensions at end systole
LVPWd LV: posterior wall thickness in diastole
LVPWs LV: posterior wall thickness in systole
RBC: Red blood cell
Hb: Hemoglobin
Ht: Hematocrit
T-cho: Total cholesterol
mTOR: mammalian target of rapamycin
PKB: protein kinase B
PPAR-α/β/δ: peroxisome proliferator-activated receptor-alpha/beta/gamma
SAS: sleep apnea syndrome
SD: standard diet;
SDS-PAGE: sodium dodecyl sulfate polyacrylamide gel electrophoresis
TG: triglycerides
ZnPPIX: zinc protoporphyrin-9
β-AR: beta-adrenergic receptors

## 1 Introduction

Hypertension, cardiac remodeling, and other complications of SAS have been studied using rodent models of intermittent hypoxia (IH) induced by short cycles of hypoxia–normoxia [[1]], [2], [3]].

Suppression of the protein kinase B (PKB), also known as Akt, mammalian target of rapamycin (mTOR) pathway (Akt-mTOR pathway) using a proprietary IH exposure device induces autophagy in rat myocardium, and blocking this induction with an inhibitor has been reported to lead to heart failure [4]. This suggests that autophagy suppresses the heart failure (HF) development in the early stages of SAS. No other confounding factors were found in the early stages of SAS. Obesity, hyperlipidemia, diabetes, and non-alcoholic fatty liver disease (NAFLD) are thought to increase the risk of cardiovascular diseases in patients with SAS. However, the association between SAS and dyslipidemia remains controversial.

SAS correlates with diabetes, hypertriglyceridemia, increased interleukin-6 (IL-6), insulin resistance, fatty liver, liver fibrosis, and inflammation [5], high density lipoprotein-cholesterol (HDL-C), but not with cholesterol, triglycerides (TG), or low-density lipoprotein-C (LDL-C) [6]. It is associated with elevated C-reactive protein (CRP) levels, insulin resistance, hepatitis, ballooning, and fibrosis, but not fatty liver [7]. We previously reported that lipid accumulation in the liver after HFD loading improved after IH exposure [8].

HFD has been thought to promote the heart failure due to pressure loading and ischemia-reperfusion, but recently, the effect of HFD on the heart failure has been attracting attention [9]. After HFD loading, peroxisome proliferator-activated receptor-alpha/beta/gamma (PPAR-α/β/δ) transcriptional activation induces the fat-metabolizing enzyme CD36 and suppresses the onset of the heart failure in a pathological model [[10], [11], [12]]. Conversely, in diabetes/obesity models (STZ, Kir6.2, ob/ob), fatty acid synthase decreased, L-type Ca2 + channel activity increased, and Ca2+/Calmodulin-Dependent Protein Kinase II (CaMKII) activated immediately after pressure loading and sudden death due to arrhythmia occurred [13].

Fatty acid metabolism is enhanced by acute pressure loading and beta-adrenergic receptors (β-AR) stimulation, but fatty acid metabolism disorders are less in the early stages of the heart failure and become more prominent as heart failure progresses, and inhibitors of fatty acid-metabolizing enzymes, such as CPT1, are useful for treating the heart failure [14].

SAS has been implicated in increased risk of heart disease, including arrhythmias and ischemic heart disease [[15], [16], [17], [18], [19]].

Obesity caused by HFD leads to the accumulation of fat in the cardiovascular system, changes metabolism, and affects the heart function. Obesity and lipid abnormalities are associated with the onset of SAS, which is associated with intermittent hypoxia.

SAS and dyslipidemia were recapitulated by IH and a HFD, respectively, in rats. It is known that IH causes hemoconcentration, and dyslipidemia increases blood viscosity, both of which are risk factors for thrombus formation.

It remains unclear whether SAS affects cardiac function in patients with dyslipidemia, and whether dyslipidemia promotes thrombosis.

The purpose of this study was to investigate the effects of IH exposure on cardiac function after HFD loading.

## 2 Materials and methods

### 2.1 Animal model

All experiments were performed in accordance with the Guide for the Care and Use of Laboratory Animals published by the US National Institutes of Health (NIH publication 85–23, revised 1996). All experiments performed in this study were approved by the Experimental Animal Committee of Tokyo Medical University (approval numbers: H310009, R2-0127, and R3-0042).

Male 5-week-old Sprague–Dawley rats were divided into two groups according to the diet they consumed: the standard diet (SD) and HFD groups. After 2 weeks, they were further divided into two groups based on IH exposure: the IH-exposed and non-exposed groups. The group that consumed the SD and was exposed to IH was the SD + IH group, and the group that consumed the HFD and was exposed to IH was the HFD + IH group. The SD group received a diet comprising 55.3% nitrogen-free extracts, 23.1% proteins, and 5.1% fats, while the HFD group's diet comprised 25.3% nitrogen-free extracts, 23.0% proteins, and 35% fats. Additionally, the SD and IH + HFD groups received a zinc protoporphyrin-9 (ZnPPIX) (Cayman Chemical, Ann Arbor, MI, USA) as a heme oxygenase-1 (HO-1) inhibitor. They were designated as the SD + ZnPPIX group and IH + HFD + ZnPPIX group, respectively. The rats were placed next to cages with IH settings at a rate of 20 cycles/h (minimum and maximum O2 concentration: 4% and 21%, respectively; CO2 concentration, 0%) or room air breathing (control). They were placed in the same plastic cage in which they were received. The IH device was functional for 8 h/day during a 12-h “light on” period. The IH exposure was for 2 weeks. The HFD rats consumed an HFD, which consisted of 25.3% proteins and 35% fats. The IH rats had free access to food and water, whereas the amount of food for the control rats was restricted weekly and adjusted to match their average body weight [4].

ZnPPIX (25 mg/kg) was administered intraperitoneally immediately before and 1 week after IH exposure.

**Fig. 1:**
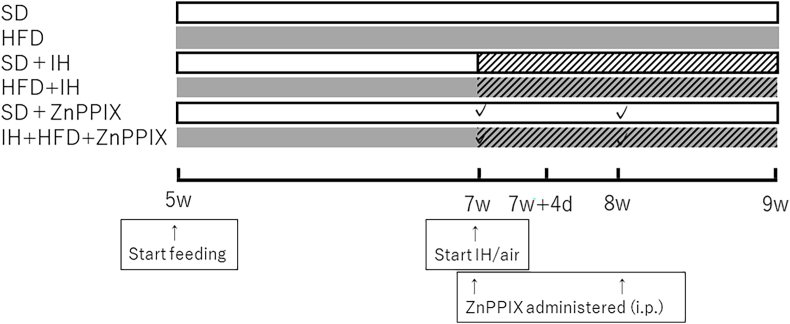
Protocol for the animal experiments. Feeding rats with a SD (white column) or a HFD (gray column) was started 2 weeks before the onset exposure to normal air or IH air for 4 days (7 weeks + 4 days old) or 2 weeks (9 weeks old). ZnPPIX was administered at 25 mg/kg immediately prior to IH exposure (7 weeks of age) and 1 week after (8 weeks of age).

These protocols are shown in Fig. 1.

### 2.2 Echocardiographic studies

Echocardiography was performed under inhalational anesthesia with 2.5 L/min isoflurane (Mylan Inc., Tokyo, Japan) using Noblus (Hitachi Ltd., Tokyo, Japan). A 2D parasternal short-axis view of the left ventricle (LV) was obtained at the level of the papillary muscles. When suitable images were obtained on the axis, 2D targeted M-mode tracings were recorded. Two consecutive heartbeats in each frame were analyzed to measure the heart rate (HR), LV anterior wall thickness in diastole (LVAWd) and systole (LVAWs), LV posterior wall thickness in diastole (LVPWd) and systole (LVPWs), LV internal dimensions at end diastole (LVDd), and LV internal dimensions at end systole (LVDs). LV fractional shortening (FS) was calculated as LVDd minus LVDs normalized for LVDd as an index of LV systolic performance. We confirmed very small intra-and inter-observer variability, along with high reproducibility, in the same animals as those used in our echocardiographic measurements.

### 2.3 Western blot analysis

The homogenized proteins from the frozen hearts were resolved by sodium dodecyl sulfate polyacrylamide gel electrophoresis (SDS-PAGE, 15% gel) and subjected to Western blot analysis. The blots were probed with one of the following primary antibodies: HO-1 (ab13248) and GAPDH (ab8245) (all from Abcam, Richmond, Canada). For chemiluminescence detection, peroxidase-conjugated anti-mouse IgG or anti-rabbit IgG (Promega, Madison, WI, USA) was used as the secondary antibody. Band densities were measured using an ImageQuant™ LAS 4000 mini (GE Healthcare UK Ltd., Buckinghamshire, England).

### 2.4 Hematology and blood biochemistry

The blood collected was quickly divided into two evacuated blood collection tubes. One used Veneject II vacuum blood collection tubes (TERUMO INC., Tokyo, Japan) containing ethylenediaminetetraacetic acid (EDTA)-2NA for hematology analysis. The other used Insepac II (Sekisui Medical Co., Ltd., Tokyo, Japan) containing rapid clotting agents (thrombin and silica) for analysis of blood biochemistry. Hematological analyzes measured red blood cell (RBC) count, hemoglobin (Hb) concentration, and hematocrit (Ht) level. Blood biochemical analyses measured Total cholesterol (T-cho), TG, and LDL-C level. These analyzes were performed by Kotobiken Medical Laboratories (Tokyo, Japan).

### 2.5 Measurement of blood fluidity

The measurement of blood fluidity with an MC-FAN (MC Healthcare Inc., Tokyo, Japan) rheometer was the same as that described by Kikuchi's microchannel method [20,21]. Briefly a V-shaped groove (width, 7 μm; length, 30 μm; depth, 4.5 μm) was made on a 15 × 15 × 0.5 mm silicon single crystal substrate of an integral circuit, using an anisotropic etching technique. Blood samples were collected via cardiac puncture under anesthesia. Heparin solution (1000 IU/mL, 0.1 mL) was added to 2 mL of the collected blood samples and used for the measurement.

### 2.6 Statistical analysis

Group data are expressed as mean ± standard error of the mean. Data were analyzed using Dunnett's test, Tukey–Kramer test, or Student's t-test at a confidence level of 95%. One-way analysis of variance was used for statistical comparisons of the recorded observational data, followed by pairwise post hoc comparisons. Statistical analysis was performed in GraphPad Prism 6 for Windows, version 6.05 (GraphPad Software, San Diego, CA, USA).

## 3 Results

### 3.1 Influence of IH and HFD loading on hematology, body weight, and the heart weight

Compared to control rats, rats exposed to high-fat load and IH for 4 days were more prone to mild weight loss. In addition, the heart weight decreased. There was no change in the heart weight relative to the body weight. HFD load and IH exposure resulted in increased RBC count, Hb concentration, and Ht levels, reflecting polyerythemia due to systemic hypoxia (Table 1). T-cho was predominantly increased in the HFD + IH + ZnPPIX group compared to that in the control rats. TG levels were not significantly different from those in the control group. LDL-C levels were significantly elevated in the HFD, HFD + IH, and HFD + IH + ZnPPIX groups (Table 1).

**Table 1:** Influence of IH 4 days on hematology, body weight, and the heart weight.

	SD n = 8	HFD n = 6	SD＋IH n = 8	HFD + IH n = 15	SD＋ZnPPIX n = 4	HFD + IH + ZnPPIX n = 6
HW(g)	1.0 ± 0.039	0.92 ± 0.028	0.91 ± 0.041	0.85 ± 0.023**	0.87 ± 0.023	0.94 ± 0.11
BW(g)	278.8 ± 6.22	251.2 ± 10.2**	249.1 ± 6.67**	230.9 ± 4.18**	258.5 ± 1.94*	232.5 ± 10.2**
HW/BW(‰)	3.5 ± 0.12	3.7 ± 0.12	3.7 ± 0.15	3.7 ± 0.074	3.4 ± 0.077	4.0 ± 0.28
RBC( × 10 μL^−4^）	661 ± 20.2	655 ± 18.7	741 ± 14.8	746 ± 20.5*	699 ± 4.77	824 ± 47.5**
Hb(g/dL)	13.4 ± 0.343	13.4 ± 0.332	14.6 ± 0.292	15.6 ± 0.449**	14.4 ± 0.0764	17.4 ± 1.15**
Ht(%)	44.6 ± 1.32	42.6 ± 1.40	46.8 ± 1.03	49.5 ± 1.15*	44.6 ± 0.525	52.9 ± 3.10*
T-cho(mg/dL)	63.5 ± 3.41	74.2 ± 3.08	63.9 ± 3.85	76.0 ± 5.51	52.5 ± 4.03	77.5 ± 3.52*
TG(mg/dL)	114 ± 9.45	149 ± 21.5	82.3 ± 11.4	91.9 ± 5.99	90.3 ± 10.1	82.8 ± 32.3
LDL-C(mg/dL)	8.13 ± 0.581	12.8 ± 0.792*	8.00 ± 0.535	11.7 ± 0.796*	10.0 ± 1.08	14.0 ± 1.41*

*p < 0.01vs. SD, **p < 0.01vs. SD. Data are meam±SEM.

At 2 weeks of IH exposure, weight loss was observed in IH-exposed rats compared to control rats. In addition, the heart weight decreased. The change in the heart weight relative to the body weight increased in the HFD + IH + ZnPPIX group. HFD load and IH exposure resulted in increased RBC count, Hb concentration, and Ht levels, reflecting polycythemia due to systemic hypoxia.

T-cho and TG levels were not significantly different among the groups. LDL-C levels were significantly elevated in the HFD, HFD + IH, and HFD + IH + ZnPPIX groups (Table 2).

**Table 2:** Influence of IH for 2 weeks on hematology, body weight, and the heart weight.

	SD	HFD	SD＋IH	HFD + IH	SD＋ZnPPIX	HFD + IH + ZnPPIX
	n = 9	n = 6	n = 5	n = 4	n = 4	n = 6
HW(g)	1.0 ± 0.029	1.1 ± 0.038	0.85 ± 0.036**	0.86 ± 0.038**	1.1 ± 0.023	0.91 ± 0.017*
BW(g)	355 ± 3.62	369 ± 3.96	286 ± 7.43**	260 ± 8.21**	346 ± 15.8	261 ± 4.77**
HW/BW(‰)	2.9 ± 0.071	3.0 ± 0.094	3.0 ± 0.086	3.3 ± 0.13*	3.2 ± 0.13	3.5 ± 0.11**
RBC( × 10 μL^−4^）	718 ± 12.7	714 ± 29.7	815 ± 11.1*	873 ± 26.0**	730 ± 33.8	861 ± 18.9**
Hb(g/dL)	14.0 ± 0.440	15.0 ± 0.621	15.8 ± 0.259*	17.7 ± 0.0500**	15.0 ± 0.636	17.5 ± 0.47**
Ht(%)	43.3 ± 2.06	49.4 ± 2.33*	47.9 ± 0.552	53.8 ± 0.132**	44.9 ± 1.38	53.6 ± 1.25**
T-cho(mg/dL)	62.6 ± 2.93	74.5 ± 6.97	54.8 ± 3.47	73.5 ± 2.90	63.3 ± 4.33	63.3 ± 5.23
TG(mg/dL)	106 ± 10.3	190 ± 34.4*	82.2 ± 13.8	69.5 ± 22.4	75.3 ± 8.22	78.8 ± 13.8
LDL-C(mg/dL)	9.11 ± 0.772	15.0 ± 2.35**	6.00 ± 0.707	15.8 ± 1.25**	8.25 ± 1.03	13.8 ± 1.19*

*p < 0.01vs. SD, **p < 0.01vs. SD. Data are meam±SEM.

### 3.2 LV function monitor for echocardiography

Echocardiographic data of 4 days IH exposure (Table 3) showed a marked increase in FS in the HFD + IH and HFD + IH + ZnPPIX groups. In addition, narrowing of the left ventricular cavity was observed during contraction. The thickness of the anterior wall of the left ventricle did not change during systole and diastole, but the posterior wall thickened in the HFD + IH and HFD + IH + ZnPPIX groups during systole. The left ventricular heart rate increased in the HFD, SD + IH, and HFD + IH groups.

**Table 3:** Influence of IH for 4 days on Echocardiographic studies.

	SD	HFD	SD＋IH	HFD + IH	SD＋ZnPPIX	HFD + IH + ZnPPIX
	n = 17	n = 19	n = 20	n = 42	n = 13	n = 18
FS(%)	54.7 ± 0.376	54.9 ± 0.401	54.4 ± 0.288	72.3 ± 1.12**	54.1 ± 0.263	72.3 ± 2.05**
LVAWd (mm)	1.60 ± 0.0354	1.58 ± 0.0280	1.58 ± 0.0278	1.65 ± 0.0277	1.45 ± 0.0427	1.55 ± 0.0442
LVAWs (mm)	2.98 ± 0.0591	2.96 ± 0.0339	2.90 ± 0.0592	3.41 ± 0.0629**	2.80 ± 0.0500	3.43 ± 0.0837**
LVDd/BW (mm/100 g)	2.65 ± 0.0500	2.78 ± 0.0379	2.80 ± 0.0413	2.71 ± 0.0403	2.85 ± 0.0677	2.73 ± 0.0842
LVDs/BW (mm/100 g)	1.20 ± 0.0292	1.26 ± 0.0228	1.28 ± 0.0243	0.771 ± 0.0361**	1.32 ± 0.0381	0.645 ± 0.0592**
LVPWd (mm)	1.93 ± 0.0366	1.92 ± 0.0531	1.89 ± 0.0475	1.95 ± 0.0413	1.78 ± 0.0537	1.86 ± 0.0479
LVPWs (mm))	3.15 ± 0.0436	3.09 ± 0.0634	3.04 ± 0.0486	3.47 ± 0.0381**	2.95 ± 0.0573	3.46 ± 0.0982**
HR (bpm)	367 ± 9.90	397 ± 8.45*	406 ± 5.34**	430 ± 4.99**	384 ± 10.0	397 ± 9.36

*p < 0.01vs. SD, **p < 0.01vs. SD. Data are meam±SEM.

Echocardiographic data of 2 weeks IH exposure (Table 4) showed a significant increase in FS in the HFD + IH + ZnPPIX group alone. Additionally, narrowing of the left ventricular cavity was observed. The anterior and posterior walls of the left ventricle thickened only in the HFD + IH + ZnPPIX group during contraction and diastole. The heart rate increased in the SD + IH, HFD + IH, and HFD + IH + ZnPPIX groups.

**Table 4:** Influence of IH for 2 weeks on Echocardiographic studies.

	SD	HFD	SD＋IH	HFD + IH	SD＋ZnPPIX	HFD + IH + ZnPPIX
	n = 9	n = 16	n = 12	n = 18	n = 4	n = 11
FS(%)	53.6 ± 0.297	54.6 ± 0.358	54.2 ± 0.554	55.2 ± 2.97	53.6 ± 0.355	71.8 ± 2.09**
LVAWd (mm)	1.67 ± 0.0701	1.65 ± 0.0295	1.61 ± 0.0323	1.59 ± 0.0397	1.49 ± 0.109	1.43 ± 0.062
LVAWs (mm)	3.15 ± 0.0817	3.0 ± 0.0315	2.97 ± 0.0372	2.96 ± 0.0352*	2.85 ± 0.114*	3.43 ± 0.0955*
LVDd/BW (mm/100 g)	2.16 ± 0.0482	2.09 ± 0.0517	2.39 ± 0.0507	2.42 ± 0.0429	2.42 ± 0.0327	2.32 ± 0.126
LVDs/BW (mm/100 g)	1.01 ± 0.0289	0.950 ± 0.0281	1.09 ± 0.281	1.08 ± 0.0246	1.12 ± 0.0128	0.672 ± 0.0714**
LVPWd (mm)	1.98 ± 0.0745	2.08 ± 0.0527	2.02 ± 0.529	1.94 ± 0.223	1.95 ± 0.0615	1.89 ± 0.0722
LVPWs (mm))	3.23 ± 0.108	3.21 ± 0.0635	3.04 ± 0.0861	2.98 ± 0.0632	3.33 ± 0.0115	3.35 ± 0.0941
HR (bpm)	361 ± 8.74	380 ± 8.55	400 ± 11.4*	418 ± 6.42**	367 ± 12.9	424 ± 8.83**

*p < 0.01vs. SD, **p < 0.01vs. SD. Data are meam±SEM.

**Fig. 2:**
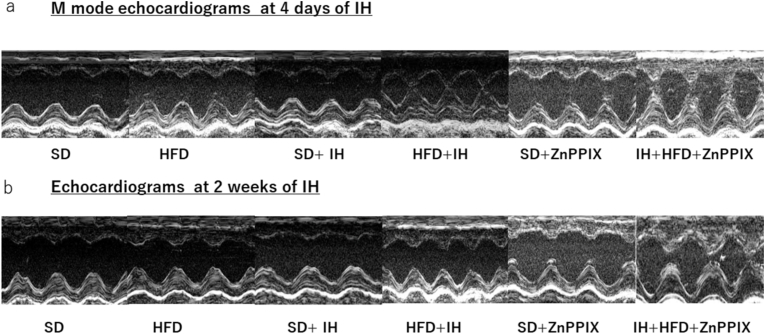
A. FS did not increase in the IH and HFD alone groups but increased with HFD loading and IH exposure. No particular effect was observed on the 4th day of ZnPPIX administration. B. Two weeks after IH exposure, FS returned to pre-IH levels in the IH + HFD group. However, this increase continued in the IH + HFD + ZnPPIX group.

Fig. 2 shows the M-mode echocardiography. Left ventricular hyperconstriction was observed in the IFD + IH and HFD + IH + ZnPPIX groups on day 4 of IH exposure (Fig. 2A), and left ventricular hyperconstriction was observed on day 2 in HFD + IH + ZnPPIX group (Fig. 2B). This is consistent with the results for FS and left ventricular stenosis in Table 3, Table 4

**Fig. 3:**
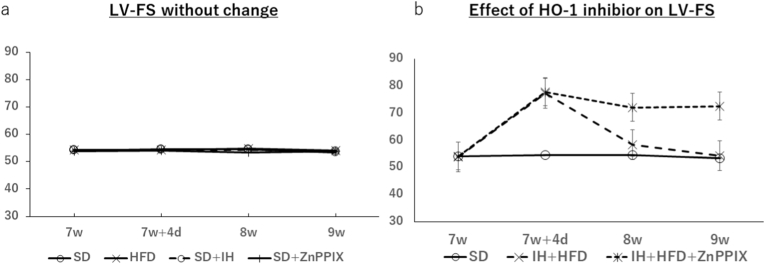
A. Left ventricular-ejection fraction (LV-EF) did not change over time in the SD, HFD, IH, and SD + ZnPPIX groups. B. However, in the IH + HFD group, LV-EF increased after peaking on the 4th day after IH exposure and gradually returned to normal 2 weeks after IH exposure. However, in the IH + HFD + ZnPPIX group, left ventricular-fractional shortening (LV-FS) remained high after IH exposure. These were recorded by echocardiography on the 4th day, and 1st and 2 nd weeks before IH exposure (each group, n = 5–43).

Changes in FS were plotted over time at 0 day, 4 days, 1 week, and 2 weeks of IH exposure (Fig. 3). No changes in FS were observed in the SD, HFD, SD + IH, or SD + ZnPPIX groups (Fig. 3A). Comparing the IH + HFD group (long dashed line) and the IH + HFD + ZnPPIX group (short dashed line) with SD as the control, the FS hypercontraction in the IH + HFD group peaked on day 4 and gradually became the same. FS hypercontraction persisted in the ZnPPIX group.

### 3.3 Western blots analysis of HO-1 in myocardium

**Fig. 4:**
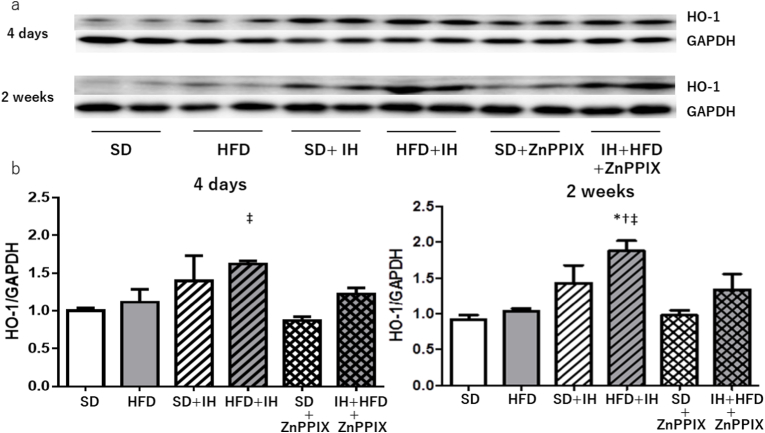
Heme oxygenase-1 (HO-1) was induced 4 days of IH in HFD fed rats, as detected by Western blot analysis. *p < 0.05vs. SD, †p < 0.01 vs. HFD, ‡p < 0.01 vs. SD + ZnPPIX. Data are meam±SEM. Each group n = 4–8.

The expression of HO-1 in the myocardium was analyzed by western blotting (Fig. 4A). HO-1 was induced 4 days after IH in HFD rats, as detected by Western blot analysis. Similar results were obtained after 2 weeks of IH exposure (Fig. 4B). The expression of HO-1 was suppressed with the administration of ZnPPIX (Fig. 4B). Administration of ZnPPIX had no effect on HO-1 expression compared with that in the SD group (Fig. 4B).

### 3.4 Measurement of blood flow rate using the MC-FAN

**Fig. 5:**
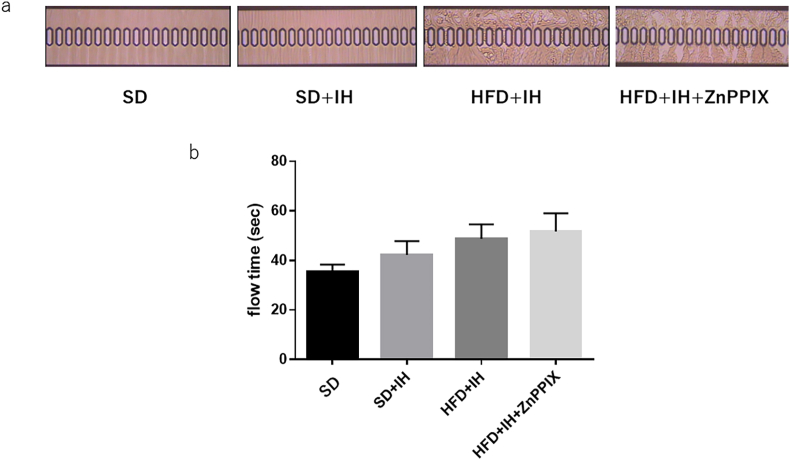
No blood flow stasis was observed in the C and IH groups, but blood flow stasis was observed in the IH + HFD and IH + HFD + ZnPPIX groups.

Blood flow was measured 2 weeks after IH exposure using the MC-FAN. It is a device that measures the time it takes for a certain amount of blood (100 μL) to pass through, by applying a certain pressure to the collected blood (whole blood), through the groove of a silicon chip that has been finely processed to the size of a capillary vessel (7 μm). Simultaneously, the state of blood cells passing through the groove of the silicon chip can be magnified approximately 2000 times with a microscope, and the image can be displayed on the monitor via the CCD (Charge Coupled Device) camera (Fig. 5A). It can be seen that blood flow is stagnant in the HFD + IH and HFD + IH + ZnPPIX groups compared to the SD group (Fig. 5A and B).

Four SD rats (SD = 4, IH = 4, HFD + IH = 4, HFD + IH + ZnPPIX = 4) were included in the study.

## 4 Discussion

There have been clinical cases of SAS and sudden death. However, the underlying mechanisms remain unclear. In addition to sleep apnea, there are several factors associated with sudden death. The present study considered sleep apnea and a HFD as factors.

To the best of our knowledge, this is the first study to explain the mechanism of IH-induced hypercontraction in an animal model loaded with an HFD. The present study explains that hypercontraction is caused by the viscosity of blood and is ameliorated by the release of HO-1. Hypercontraction continued when the release of HO-1 was suppressed by ZnPPIX.

### 4.1 Cardiac effects of IH exposure due to high fat load

IH exposure caused weight loss (Table 1, Table 2). HFD loading increased the heart weight per body weight in the second week of IH exposure. One of the causes of cardiac hypertrophy is repeated excessive contraction of the heart muscle due to high blood pressure, which is caused by the thickening of the heart muscle. Evaluation of myocardial thickness revealed that the anterior and posterior walls of the myocardium thickened during contraction on day 4 of IH exposure due to HFD loading. In the second week, thickening was seen only in the anterior wall during contraction in the HFD + IH + ZnPPIX group (Fig. 3, Fig. 4). This is consistent with the fact that the myocardium was excessively thickened during contraction due to the hypercontraction of the heart on the 4th day, but the hypercontraction was released in the 2 nd week, and at the same time, the heart muscle thickening reduced. The thickness of the diastolic ventricular wall was not significantly different between the groups on days 4 and 14 after IH exposure. Excessive contraction of the heart is represented in “Takotsubo syndrome,” caused by sudden stress. This was accompanied by partial hypercontraction of the apex of the heart [22,23]. In our model, hypercontraction peaks and gradually returns to normal 4–5 days after intermittent hypoxic exposure to a HFD. Therefore, intermittent hypoxic stress alone did not cause hypercontraction. Therefore, it differs from the commonly known stress response [24].

Hypercontraction is thought to be the pumping action that pumps blood flow throughout the body owing to stagnation of blood flow. It is widely known that hypoxia causes hemoconcentration [[25], [26], [27], [28]]. Hypercholesterolemia also reduces nitric oxide (NO) production from vascular endothelial cells and reduces vascular protective function [[29], [30], [31]], but in addition to NO production, blood viscosity elevates blood concentration, white blood cell attachment, increases platelet aggregation [32,33], and decreases tissue circulation. Hypercholesterolemia reduces the ability of red blood cells to deform [34]. In particular, changes in the lipid composition of cell membranes are known to reduce erythrocyte deformability [35,36], which is an important factor in controlling organ microcirculation. Furthermore, erythrocytes are not only affected by NO supplied from the vascular endothelium [[37], [38], [39]] but also by endothelial NO synthase (eNOS) [40,41], which is involved in microcirculation by producing NO. Experimental results also showed stagnation of blood flow due to blood concentration (Table 1, Table 2) and erythrocyte deformability (Fig. 5).

Therefore, we used MC-FAN [[42], [43], [44]] to assess the blood flow. As shown in the results, blood flow can be seen through a slit with a width of 7 μm that mimics capillaries; however, blood flow usually flows like SD or SD + IH. However, when blood flow is stagnant, blood components accumulate on the slit, such as with HFD + IH and HFD + IH + ZnPPIX, and do not flow smoothly from top to bottom (Fig. 5A). Blood flow and stagnation were observed with HFD + IH and HFD + IH + ZnPPIX (Fig. 5A and B), but echocardiography did not show hypercontraction with HFD + IH. This will be explained in the next section.

### 4.2 Release of HO-1 with vasodilatory effect

As shown in the previous section, HFD + IH at 2 weeks of IH exposure did not cause myocardial hypercontraction despite the stagnation of blood flow. This is probably due to the improved blood flow.

As shown in Fig. 4, an increase in HO-1 was observed in the HFD + IH group after both 4 and 2 weeks of intermittent hypoxic exposure. A protein called HO-1 is used to break down heme to biliverdin. Biliverdin releases iron and carbon monoxide and acts on vasodilation [45]. In addition, HO-1 expression was suppressed by ZnPPIX [46]. As shown in Fig. 4, intraperitoneal administration of ZnPPIX in the HFD + IH group inhibited HO-1 expression. Hypercontraction persisted without vasodilation owing to HO-1 inhibition (Fig. 3B). Regarding blood flow, as explained in the previous section, stagnation occurred in the HFD + IH and HFD + IH + ZnPPIX groups during the second week of intermittent hypoxic exposure. In other words, there was a delay in the blood flow time in both groups, and echocardiography did not cause myocardial hypercontraction in the HFD + IH group, but it did cause myocardial hyperconstriction in the HFD + IH + ZnPPIX group. The results of this experiment showed that vasodilation improved blood flow. This is considered to be due to the biocompatibility of the individual.

Heme oxygenase is induced by hemodynamic forces of the vascular smooth muscle and endothelial cells. Using the mesenteric artery ligation model [47], Mohamed Lamine Freidja et al. reported the involvement of HO-1 in flow (shear force) dependent remodeling using the mesenteric artery ligation model [47]. It depends on NO produced by shear stress and mitochondrial hydrogen peroxide [47]. In our experiments, we speculated that the addition of a HFD induced HO-1, stagnating red blood cell flow, and dilating blood vessels. Stagnation of blood flow can induce thrombosis but was not found during the observation period.

### 4.3 Clinical significance

In patients with SAS, sleep apnea attacks are said to cause sudden cardiac death due to myocardial infarction and heart failure [[48], [49], [50]]. As SAS may be associated with a variety of underlying disorders, we also investigated its relationship with a HFD. Experiments have shown that the combination of hypoxia, which affects blood levels, and a HFD, which interferes with the ability of red blood cells to deform, causes hypercontraction of the heart, resulting in the release of HO-1. Dilation of the blood vessels normalizes cardiac function. However, if the release of HO-1 is blocked, hypercontraction continues. Hypercholesterolemia not only weakens blood vessels, but also affects cardiac function when hypoxia is present. Persistent hypercontraction can cause cardiac hypertrophy. However, the heart function of patients with hypercholesterolemia appears normal.

HO-1 may be released by biological adaptation, suggesting the need for treatment of the underlying hypercholesterolemia.

## 5 Summary

Intermittent hypoxic exposure in a rat model of intermittent hypoxia supplemented with a HFD causes peak myocardial hyper constriction on days 4–5. Myocardial hypercontraction is caused by an increase in the blood viscosity. The heart contracts strongly, pumping viscous blood to every corner of the body and causing myocardial hypercontraction. Therefore, HO-1 is released as part of a biological defense mechanism. HO-1 has a vasodilatory effect and suppresses hyper contractions. Therefore, IH exposure to HFD causes and promotes transient hyper constriction, releasing HO-1 as a biological response. We conclude that the blood vessels were dilated, and the EF returned to normal. However, it needs to be stressed that even if EF returns to normal levels, it is not a radical cure.

## 6 Novelty and significance

### 6.1 What is known

・Obesity, hyperlipidemia, diabetes, and non-alcoholic fatty liver disease (NAFLD) are thought to increase the risk of cardiovascular diseases in patients with SAS. However, the association between SAS and dyslipidemia remains controversial.
・HFD has been thought to promote heart failure due to pressure loading, ischemia-reperfusion, etc., but recently, the heart failure-suppressing effect of HFD has been attracting attention.

### 6.2 “What new information does this article contribute?

Rats model of IH supplemented with a HFD causes cardiac hyper constriction in a few days, but the release of HO-1 dilates blood vessels and suppresses cardiac hypercontraction. Apparently, EF returns to normal, but it is not a radical cure.

## Sources of funding

This research was supported by the Grant-in-Aid for Scientific Research (C; 19K10694) from the Japan Society for the Promotion of Science (JSPS). The technical support for this research was given by the Animal Research Center of Tokyo Medical University.

## Declaration of competing interest

The authors declare that they have no known competing financial interests or personal relationships that could have appeared to influence the work reported in this paper.

## Data Availability

No data was used for the research described in the article.
